# Acknowledgement to the Reviewers of GMS Journal for Medical Education

**DOI:** 10.3205/zma001078

**Published:** 2017-02-15

**Authors:** Martin R. Fischer, Götz Fabry

**Affiliations:** 1 GMS Journal for Medical Education, Chief Editor, Erlangen, Germany; 2 Klinikum der Universität München, Institut für Didaktik und Ausbildungsforschung in der Medizin, München, Germany; 3 GMS Journal for Medical Education, Assistant Chief Editor, Erlangen, Germany; 4 Albert-Ludwig-Universität Freiburg, Medizinische Fakultät, Abteilung für Medizinische Psychologie und Soziologie, Freiburg, Germany

## Acknowledgement

We gratefully acknowledge the valuable contribution of the following peer reviewers, who supported our Journal in 2016. We are looking forward to continuing our collaboration!

## Reviewers in alphabetical order

- Harald Affeldt, Mainz
- Sven Anders, Hamburg
- Matthias Angstwurm, München
- Sebastian Arlt, Berlin
- Cadja Bachmann, Hamburg
- Katrin Balzer, Lübeck
- Franziska Bässler, Heidelberg
- Daniel Bauer, Bern
- Nicola Bauer, Bochum
- Erika Baum, Marburg
- Yasmin Bayer, Würzburg
- Jan Becker, Münster
- Pascal Berberat, München
- Christoph Berendonk, Bern
- Sarah Berger, Heidelberg
- Mathias Berger, Freiburg
- Silke Biller, Basel
- Martin Boeker, Freiburg i. Br.
- Klaus Böhme, Freiburg i. Br.
- Hans Martin Bosse, Düsseldorf
- Jan Breckwoldt, Zürich
- Beate Brem, Bern
- IreneBrunk, Berlin
- Heinz Bruppacher, Zürich
- Jean-Francois Chenot, Greifswald
- Renate Deinzer, Gießen
- Peter Dieter, Dresden
- Konstantin Dimitriadis, München
- Marcus G. Doherr, Berlin
- Markus Dürsch, Göttingen
- Friedrich Edelhäuser, Witten
- Claudia Ehlers, Jena
- Jan P. Ehlers, Witten
- Maren Ehrhardt, Hamburg
- Nurith Epstein, München
- Michael Ewers, Berlin
- Folkert Fehr, Sinsheim an der Elzenz
- Andreas Fichtner, Halle
- Sabine Ingrid Fischbeck, Mainz
- Volkhard Fischer, Hannover
- Andreas Fleischmann, München
- Peter Frey, Bern
- Hendrik Friederichs, Münster
- Angelika Hiroko Fritz, Essen
- Petra Ganschow, München
- Karim Gawad, Hamburg
- Kirsten Gehlhar, Oldenburg
- Waltraud Georg, Berlin
- Susanne Gerhardt-Szép, Frankfurt/Main
- Marianne Giesler, Freiburg i. Br.
- Adrian Goeldlin, Bern
- Katja Götz, Lübeck
- Matthäus Grasl, Wien
- Jan Griewatz, Tuebingen
- Christian Gruber, Hannover
- Martin Haag, Heilbronn
- Petra Hahn, Freiburg
- Eckhart G. Hahn, Erlangen
- Wolfgang Hampe, Hamburg
- Sigrid Harendza, Hamburg
- Anja Härtl, München
- Wolf Hautz, Bern
- Stefanie C. Hautz, Bern
- Richard Hays, Hobart
- Jan Hense, Gießen
- Anne Herrmann-Werner, Tübingen
- Matthias Hofer, Düsseldorf
- Anke Hollinderbäumer, Mainz
- Anita Holzinger, Wien
- Thorsten Hornung, Bonn
- Bert Huenges, Bochum
- Sören Huwendiek, Bern
- Peter Iblher, Lübeck
- Paul Jansen, Kamen-Heeren
- Jonas Johannink, Berlin
- Ingo Just, Hannover
- Sylvia Kaap-Fröhlich, Zürich
- Jens Kaden, Mannheim
- Martina Kadmon, Oldenburg
- Hanna Kaduszkiewicz, Kiel
- Janine Kahmann, Heidelberg
- Gudrun Karsten, Kiel
- Jan Kiesewetter, München
- Claudia Kiessling, Neuruppin
- Klaus Klose, Marburg
- Michael Knipper, Giessen
- Volker Köllner, Teltow
- Sarah König, Würzburg
- Sascha Köpke, Lübeck
- Felix Krause, Leipzig
- Markus Krautter, Heidelberg
- Karl-Friedrich Krey, Greifswald
- Joachim Kugler, Dresden
- Felicitas Lahner, Bern
- Ronny Lehmann, Heidelberg
- Beate Lenk, Erfurt
- Martin Lischka, Wien
- Christiane Luderer, Halle (Saale)
- Richard Maerz, Wien
- Cornelia Mahler, Heidelberg
- Maren März, Leipzig
- Andreas Möltner, Heidelberg
- Brigitte Müller-Hilke, Rostock
- Elisabeth Narciß, Mannheim
- Christoph Nikendei, Heidelberg
- Zineb Miriam Nouns, Berlin
- Dino Carl Novak, Berlin
- Frank Nürnberger, Frankfurt/Main
- Falk R. Ochsendorf, Frankfurt/Main
- Wolfgang Öchsner, Ulm
- Tim Peters, Bochum
- Harm Peters, Berlin
- Bettina Pfleiderer, Münster
- Swetlana Philipp, Jena
- Karen Pierer, Innsbruck
- Reinhard Putz, München
- Christina Quandt, Hannover
- Sabine Ramspott, München
- Oliver Rauprich, München
- Christopher Roberts, Sydney
- Katrin Rockenbauch, Leipzig
- Kim Rooney, Tasmania
- Marco Roos, Erlangen
- Miriam Rüsseler, Gießen
- Sabine Salloch, Bochum
- Thorsten Schäfer, Bochum
- Stefan Schauber, Oslo
- Jörg Schelling, München
- Theresa Scherer, Bern
- Ralf Schmidmaier, München
- Anita Schmidt, Erlangen
- Michael Schmidts, Krems
- Wolfram Schüffel
- Jobst-Hendrik Schultz, Heidelberg
- Sylvie Schuster, Basel
- Katrin Schüttpelz-Brauns, Mannheim
- Hanna Seidling, Heidelberg
- Thomas Shiozawa, Tübingen
- Matthias Siebeck, München
- Anne Simmenroth, Göttingen
- Melanie Simon, Aachen
- Sasa Sopka, Aachen
- Beat Sottas, Bourguillon
- Anke Spura, Magdeburg
- Robin Stark, Saarbrücken
- Jost Steinhäuser, Lübeck
- Bernhard Steinweg, Bonn
- Christoph Stosch, Köln
- Irmgard Streitlein-Böhme, Freiburg i. Br.
- Julia Tabatabai, Heidelberg
- Nils Thiessen, Bonn
- Christian Thrien, Köln
- Daniel Tolks, München
- Olaf van dem Knesebeck, Hamburg
- Hendrik van den Bussche, Hamburg

